# Survival outcomes in trial defined high-risk hormone receptor-positive/human epidermal growth factor receptor II-negative early breast cancer: impact of adjuvant chemotherapy

**DOI:** 10.1007/s12672-025-02601-4

**Published:** 2025-05-16

**Authors:** Ta-Chung Chao, Deanna Gracia, Chan-Heng Ho, Hao-Yang Chen, Chi-Cheng Huang, Ling-Ming Tseng

**Affiliations:** 1 Comprehensive Breast Health Center, Taipei Veterans General Hospital, Taipei, Taiwan; 2 Division of Cancer Prevention, Department of Oncology, Taipei Veterans General Hospital, Taipei, Taiwan; 3 School of Medicine, College of Medicine, National Yang Ming Chiao Tung University, Taipei, Taiwan; 4 Department of Public Health, College of Public Health, National Taiwan University, Taipei, Taiwan; 5 Division of Breast Surgery, Department of Surgery, Taipei Veterans General Hospital, Taipei, Taiwan; 6 Institute of Epidemiology and Preventive Medicine, College of Public Health, National Taiwan University, Taipei, Taiwan

**Keywords:** Early breast cancer, CDK4/6 inhibitors, Adjuvant chemotherapy, Risk stratification

## Abstract

**Background:**

Clinical trials have shown the efficacy of adding CDK4/6 inhibitors to standard endocrine therapy in hormone receptor (HR)-positive, human epidermal growth factor receptor II (HER2)-negative high-risk early breast cancer.

**Materials and methods:**

HR+ /HER2− early breast cancers were retrieved from cancer registry. The primary endpoints were overall survival (OS) and recurrence-free survival (RFS) among trial-defined high-risk patients, as well as the impact of adjuvant chemotherapy.

**Results:**

Among 2758 registered cases, 511 and 1207 patients met MonarchE (M) and NATALEE (N) high-risk criteria, respectively. OS was 94.8% for M-high/N-high, 96.8% for M-low/N-high, 90.7% for M-high/N-low, and 98.9% for M-low/N-low patients, with a hazard ratio (HR) of 2.3 and 2.8 for M-high and N-high, respectively. For RFS, chemotherapy reduced recurrence risk in M-high patients (HR: 0.24) but showed no benefit for N-high patients overall, except for stage III N-high cases (HR: 0.2).

**Conclusion:**

Adjuvant chemotherapy significantly reduced recurrence risk in M-high patients with early breast cancer. Further stratification of M-low/N-high patients is needed to guide tailored chemotherapy approaches alongside CDK4/6 inhibition.

**Supplementary Information:**

The online version contains supplementary material available at 10.1007/s12672-025-02601-4.

## Introduction

Cyclin-dependent kinases 4 and 6 (CDK4/6) are key enzymes that regulate cell division. CDK4/6 inhibitors (CDK4/6i) are designed to interrupt cancer cell proliferation by selectively inhibiting these enzymes, leading to cell cycle arrest and subsequent tumor growth inhibition [1]. When combined with endocrine therapy (ET), CDK4/6i enhance the effectiveness of ET and improves outcomes in hormone receptor-positive (HR+)/human epidermal growth factor receptor II-negative (HER2 −) breast cancer [2, 3]. These inhibitors have transformed the treatment landscape for HR+/HER2− breast cancer, demonstrating significant benefits in advanced/metastatic and early-stage settings by addressing treatment resistance and enhancing ET efficacy [1, 4–7].

Despite advances in therapy, patients with HR+ /HER2− early breast cancer (EBC) remain at risk of recurrence even after surgery, adjuvant chemotherapy and ET. Studies report a 3-year recurrence risk of 5–15% depending on lymph node involvement and disease stage [8]. The 5-year recurrence risk is approximately 12% for the overall HR+/HER2− EBC population, and the risk persists long-term, with recurrences occurring steadily up to 20 years post-treatment [9, 10]. Node-positive disease presents a particularly high risk, with 1 in 6 women experiencing recurrence or death within 5 years [9]. Factors such as lymph node involvement, tumor size, grade, and other clinical and pathological characteristics significantly influence recurrence risk [11]. Addressing this persistent risk following curative surgery, adjuvant chemotherapy, and ET is still a critical challenge in the management of HR+/HER2− EBC.

Recent phase III clinical trials, MonarchE and NATALEE, have demonstrated the efficacy of adding CDK4/6i to standard ET in trial-defined high-risk populations [12, 13]. Identifying high-risk patients who may benefit from more aggressive adjuvant therapies is crucial for improving long-term outcomes. Clinical trials like MonarchE and NATALEE have established important criteria for defining high-risk early breast cancer and demonstrated the efficacy of adding CDK4/6i to standard endocrine therapy in these populations. However, questions remain regarding the optimal use of adjuvant chemotherapy in conjunction with CDK4/6i, and the applicability of these trial-derived risk criteria in diverse real-world settings. Therefore, this study aims to utilize real-world data from a single institution in Taiwan to evaluate treatment outcomes for patients meeting the high-risk criteria defined in the MonarchE and NATALEE trials, and to investigate the impact of adjuvant chemotherapy in this patient population.

In this study, we utilized real-world data from a single institution to evaluate treatment outcomes for patients meeting the risk criteria defined in these trials. Additionally, as the necessity of prior chemotherapy before CDK4/6 inhibition remains uncertain, we also investigated the impact of adjuvant chemotherapy in this high-risk population [14].

## Materials and methods

This study was reviewed and approved by the Institutional Review Board of Taipei Veterans General Hospital (protocol number: 2024-06-007B), which granted a waiver of informed consent due to the exclusive use of anonymized data.

### Patient inclusion/exclusion criteria

This study included women diagnosed with breast cancer at Taipei Veterans General Hospital, a tertiary referral medical center in North Taiwan. Patient data were recorded in the Taiwan Cancer Registry (TCR), which includes a long-form cancer registry [15]. The long-form database provided detailed information, including the date of initial diagnosis, pathology, clinical TNM stage, surgical procedures, treatment dates, and performance status [16, 17]. Since 2011, site-specific factors (SSFs), such as estrogen and progesterone receptor status and axillary lymph node status, have been recorded. The enrollment criteria included patients with stage I to III HR+/HER2− negative EBC. Patients with incomplete follow-up or missing data regarding HR, grade, tumor size or axillary lymph node status were excluded. As Ki-67 status became mandatory only after 2018, additional sources were utilized to obtain missing Ki-67 data for earlier cases. Specifically, local cancer case management system was used to supplement Ki-67 information. Using unique patient IDs, clinical characteristics, treatment patterns, and outcomes were compiled for analysis. Patients with incomplete or unreliable Ki-67 values were excluded.

### Risk stratification of HR+/HER2− early breast cancer

Risk stratification was based on criteria from the MonarchE and NATALEE trials. The MonarchE trial defined high-risk HR+/HER2− EBC as node-positive (N+) disease with either four or more positive lymph nodes, or one to three positive nodes combined with at least one risk factor such as tumor size ≥ 5 cm, histologic grade III, or Ki-67 ≥ 20% (Cohort 2) [12, 18]. The NATALEE trial expanded high-risk criteria to include node-negative (N0) patients with grade III disease or grade II disease with a high genomic risk profile (e.g., MammaPrint/Oncotype) or Ki-67 ≥ 20% [19, 20]. Both trials aimed to reduce the risk of recurrence by identifying patients likely to benefit from additional therapy beyond standard ET. These high-risk factors guided the stratification of risk in this study.

### Outcome variables

Patients were followed for up to 80 months to compare survival outcomes across risk stratification groups and evaluate the impact of adjuvant chemotherapy. Outcome variables included overall survival (OS) and recurrence-free survival (RFS). OS was defined as the time from diagnosis to death, while RFS was the time from diagnosis to the first recurrence or death. Clinical factors such as cancer stage and grade were included as covariates in multivariable regression models.

### Statistical analysis

Categorical variables were summarized as counts and percentages, and their distributions were compared using the Chi-Square test. A two-sided P-value of < 0.05 was considered statistically significant. Survival periods were estimated using the Kaplan–Meier method and compared using the log-rank test. Multivariable analysis was conducted using the Cox proportional hazards model, with statistical significance set at P < 0.05. Survival times beyond 80 months were right-censored.

## Results

### Risk group distributions

A total of 2758 HR+/HER2− EBC patients with complete clinical data were included in the study. The distribution of pathological stages based on the 8th AJCC staging system was as follows: IA (45.8%, n = 1263), IIA (28.9%, n = 798), IIB (10.8%, n = 297), IIIA (7.5%, n = 207), IIIB (1.1%, n = 30), and IIIC (5.9%, n = 163) [21]. Tumor size distributions were T1 (58%), T2 (35%), T3 (2%), and T4 (1%), while nodal status was N0 (66%), N1 (21%), N2 (7%), and N3 (6%). Among these patients, 18.5% (n = 511) and 43.8% (n = 1207) met the high-risk criteria for MonarchE and NATALEE trials, respectively.

### Overall survival

The mean OS was 5.6 years (SD: 0.4) for NATALEE high-risk (N-H) patients and 6.7 years (SD: 0.1) for NATALEE low-risk (N-L) patients, with the median survival not reached. At 8 years, the OS rate was 96% for N-H patients and 98.7% for N-L patients, with a hazard ratio (HR) of 2.3 (95% CI 1.4–3.8) for N-H.

**Fig. 1:**
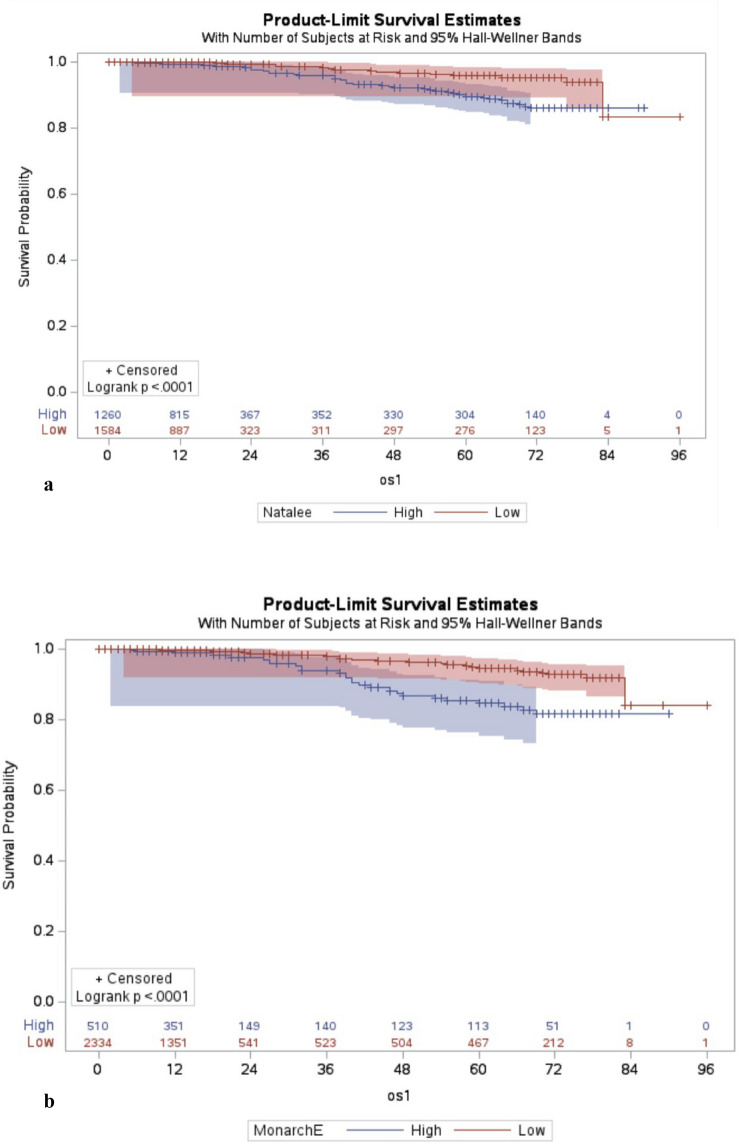
**A**, **B** Overall survival of the high- and low-risk groups defined by the NATALEE (**A**) and MonarchE (**B**) trials. The defined risk groups show a significant overall survival discrepancy (log-rank test: P = 0.001 and P < 0.001 for the NATALEE and MonarchE risk definition, os1: overall survival, *Y*-axis: survival time in months)

For MonarchE high-risk (M-H) patients, the mean survival was 5.3 years (SD: 0.1) compared to 6.7 years (SD: 0) for MonarchE low-risk (M-L) patients. The HR for M-H patients was 2.8 (95% CI 1.8–4.5) compared to the M-L group. Figure 1A and B illustrate OS stratified by both trial criteria, demonstrating strong prognostic value in the Taiwanese population.

**Fig. 2:**
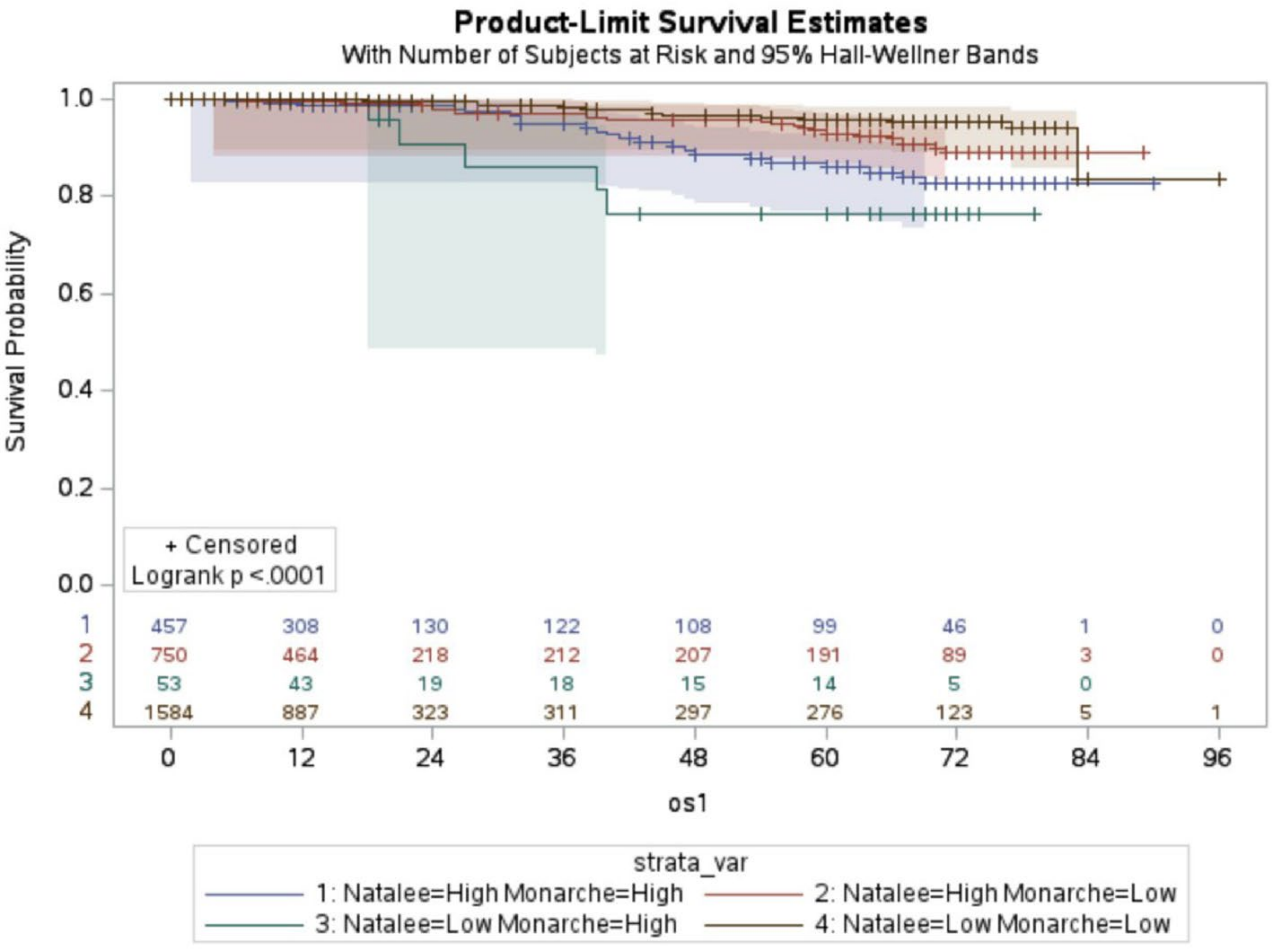
Overall survival for breast cancer risk groups defined by the NATALEE (N) and MonarchE (M) trial criteria. Patients were categorized into N-H/M-H, N-H/M-L, N-L/M-H, and N-L/M-L risk groups (log-rank test: P < 0.001, os1: overall survival, *Y*-axis: survival time in months)

When stratifying patients by both trial risk groups, 16% (n = 457) were N-H/M-H, 26% (n = 750) were N-H/M-L, 2% (n = 53) were N-L/M-H, and 56% (n = 1584) were N-L/M-L. The corresponding 8-year OS rates were 94.8%, 96.8%, 90.6%, and 98.9%, with mean survival times of 5.4, 5.7, 3.1, and 6.7 years, respectively (Fig. 2, median not reached).

**Fig. 3:**
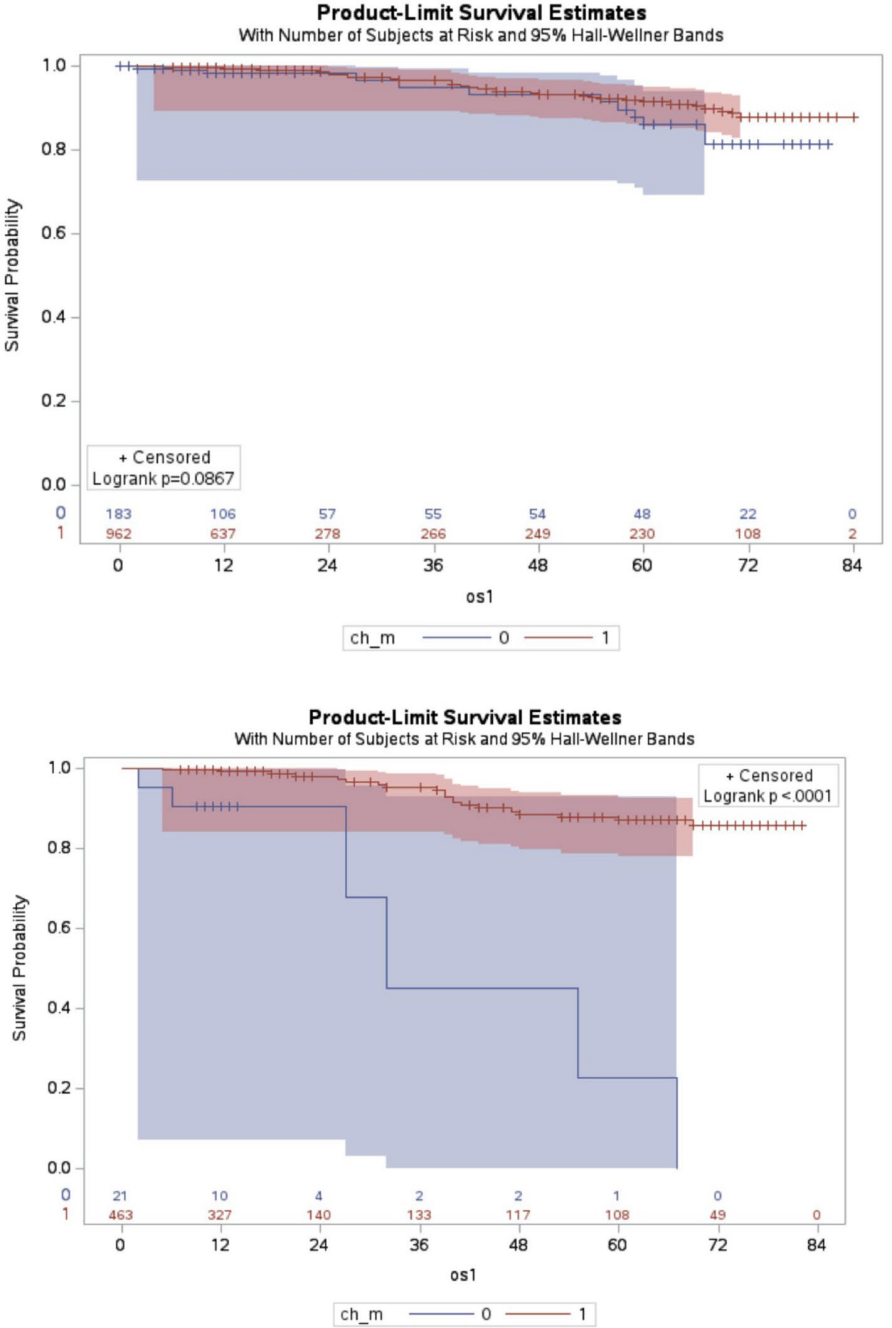
**A**, **B** Overall survival of the high-risk breast cancers defined by the NALATEE (**A**) and the MonarchE trial (**B**) with and without chemotherapy (ch_m: chemotherapy, os1: overall survival, *Y*-axis: survival time in months)

The proportion of patients receiving adjuvant chemotherapy increased with stage: 34.9% for stage I, 71.9% for stage II, and 96.1% for stage III. Adjuvant chemotherapy improved OS for N-H patients from 93.4% to 96.6%, though this was not statistically significant (log-rank test: P = 0.09). For M-H patients, chemotherapy significantly improved OS from 71.4% to 95.5% (log-rank test: P < 0.001; Fig. 3A and B).

### Recurrence-free survival

**Fig. 4:**
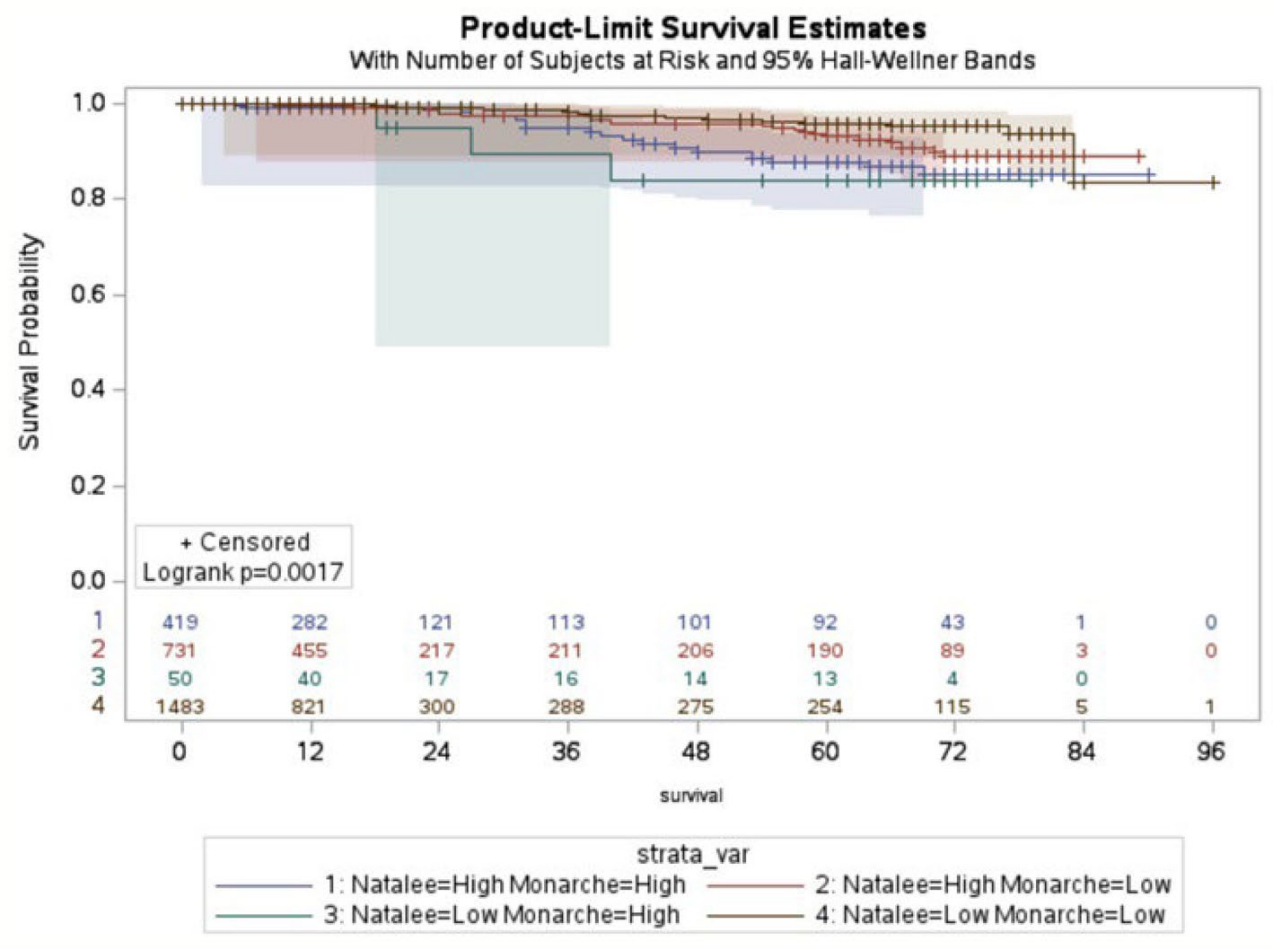
Recurrence-free survival for breast cancer risk groups defined by the NATALEE (N) and the MonarchE (M) trial criteria. Patients were categorized into N-H/M-H, N-H/M-L, N-L/M-H, and N-L/M-L risk groups (log-rank test: P = 0.017, *Y*-axis: survival time in months)

Pathological stage was a key prognostic factor for recurrence-free survival (RFS): HR for stage II vs I was 2.0 (95% CI 1.2–3.3), and HR for stage III vs I was 5.4 (95% CI 3.2–9.0; Supplementary Fig. 1). The 8-year recurrence-free rates were 98.2%, 96.3%, and 90.2% for stages I, II, and III, respectively. For N–H/M-H, N–H/M-L, N-L/M-H, and N-L/M-L patients, the 8-year RFS rates were 95.5%, 96.9%, 94%, and 98.9%, with mean RFS of 5.4, 5.7, 3.2, and 6.7 years, respectively (Fig. 4). The median RFS was not reached.

Adjuvant chemotherapy had a significant impact on M-H patients, reducing the HR for recurrence to 0.2 (95% CI 0.1–0.5, P = 0.0002) and improving the recurrence-free rate from 81.3% to 93.3%. However, for N-H patients, the HR was 1.0 (95% CI 0.6–1.7), with recurrence-free rates of 94.5% with chemotherapy and 96% without. A post-hoc subgroup analysis revealed that only stage III N-H patients derived significant benefit from chemotherapy, with an HR of 0.2 (95% CI 0.1–0.7) and an improvement in recurrence-free rate from 72.7% to 92.4% (log-rank test: P = 0.004). Supplementary Table 1 summarizes main survival outcomes and key findings for clarity and accessibility.

## Discussion

The clinical application of CDK4/6i has expanded significantly from their initial use in advanced/metastatic breast cancers to early-stage disease settings. Initially, CDK4/6i was used to manage HR+/HER2− metastatic breast cancer, where they demonstrated a remarkable ability to halt disease progression and prolong survival when combined with ET [22]. More recently, their use has extended to EBC, particularly in the adjuvant setting, to prevent recurrence by targeting residual microscopic disease. In these settings, CDK4/6i complement standard therapies to improve long-term outcomes. Clinical trials have provided strong evidence for their effectiveness in reducing relapse risk in patients with high-risk EBC [23].

In this study, we evaluated OS and RFS among a cohort of Taiwanese HR+/HER2− EBC patients treated with contemporary therapies. Among these patients, 18% met the M-H criteria, while 42% were categorized as N-H. Survival discrepancies were observed between the defined risk groups, with hazard ratios of 2.3 and 2.8, respectively, indicating the prognostic power of these stratifications. Our findings suggest that more than 40% of Taiwanese high-risk HR+/HER2− EBC patients could benefit from adjuvant CDK4/6i. The higher hazard ratio for the M-H group highlights a relatively higher baseline risk defined by the MonarchE criteria compared to NATALEE criteria.

When combining both criteria, we found that 56% of patients were categorized as low risk, 16% as concurrently high risk (N-H/M-H), and 28% as discordant risk (e.g., N-H/M-L or N-L/M-H). The worst overall survival was observed in the N-L/M-H subgroup (2%), while M-H patients consistently exhibited poorer survival outcomes regardless of NATALEE-defined risk. These results underscore the robust prognostic power of the MonarchE criteria, which may utilize a stricter risk definition. On the other hand, pT1N1micro (stage IB) patients, once with grade III or Ki67 ≥ 20% status, were destined to be N-L/M-H, while this worst minority deserves further evaluation with more cased enrolled.

It is widely accepted that adjuvant chemotherapy should generally precede the use of CDK4/6i in high-risk HR+/HER2− EBC. Chemotherapy addresses aggressive disease features, while CDK4/6i, such as abemaciclib or ribociclib, in combination with ET, suppresses residual cancer proliferation to reduce relapse risk [13, 24, 25]. Despite the better tolerability of CDK4/6i compared to chemotherapy, they cannot yet fully replace chemotherapy in the adjuvant setting for high-risk patients. For M-H patients in our study, chemotherapy significantly improved overall survival from 71.4% to 95.5%. Conversely, for N-H patients, the survival benefit was modest and statistically nonsignificant (93.4% to 96.6%), further underscoring the elevated baseline risk in M-H patients.

Analysis of RFS patterns revealed that the worst outcomes were observed in the N-L/M-H subgroup (94%), followed by N-H/M-H (95.5%). In contrast, M-L patients demonstrated better recurrence-free survival regardless of NATALEE risk status (96.9%-98.9%). These findings suggest that the MonarchE criteria may offer a more robust framework for risk stratification in Taiwanese HR+/HER2− EBC patients. Adjuvant chemotherapy remains crucial for M-H patients due to their high risk of recurrence and death, which can be improved significantly with chemotherapy. However, for some N-H patients, chemotherapy may be omitted without significantly compromising outcomes. For example, more N-H patients remained recurrence-free without chemotherapy compared to M-H patients who received chemotherapy (94.5% vs. 81.3%).

A post-hoc analysis revealed that, within the N-H group, only pathological stage III patients benefited significantly from chemotherapy, with RFS improving from 81.3% to 93.3% (hazard ratio: 0.2). Pathological stage remains a key prognostic factor for recurrence, with survival rates decreasing from 98.2% (stage I) to 90.2% (stage III). Chemotherapy was commonly administered across stages I, II, and III (34.9%, 71.9%, and 96.1%, respectively). Among stage III patients classified as both N-H and M-H, chemotherapy improved recurrence-free survival from 78.6% to 94% (hazard ratio: 0.2; 95% CI 0.1–0.5; P = 0.009), though the primary driver of this benefit may have been the M-H population.

Chemotherapy remains indispensable for addressing micrometastatic disease, especially in patients with high-risk features. CDK4/6i is increasingly used in adjuvant therapy, often following chemotherapy, to improve disease control while minimizing the adverse effects typically associated with chemotherapy [5]. Although CDK4/6i offers a favorable toxicity profile, it currently serves as a complementary rather than a replacement therapy in adjuvant settings. In addition, higher serum levels of trace elements like selenium and zinc are associated with improved cancer survival, including in breast cancer, due to their roles in immune function, antioxidant defense, and apoptosis. However, both deficiencies and excesses can be harmful, highlighting the need for balanced levels [26].

The NATALEE and MonarchE trials used different criteria for defining high-risk HR+/HER2− EBC patients. The M-H group consistently demonstrated elevated recurrence rates, underscoring the necessity of adjuvant chemotherapy. The N-H criteria, which target a broader population, often include M-H patients, though the reverse is not always true. Multi-gene expression assays, such as Oncotype DX and Mammaprint, further inform risk stratification by providing insights into recurrence risk and chemotherapy benefit [27–32]. These tests may refine treatment plans, particularly in the context of CDK4/6i, potentially sparing some patients from unnecessary chemotherapy. Our study suggests that all M-H and stage III N-H patients should receive chemotherapy before initiating CDK4/6i.

To explore why MonarchE criteria predict poorer outcomes compared to NATALEE, a more detailed analysis is needed, focusing on the nuanced differences in risk factor definitions between the trials (as shown in Table 1). This should involve a granular breakdown of individual risk factors within each cohort, such as the compositions of specific nodal involvement, tumor size, grade, and Ki-67 index. MonarchE focuses heavily on nodal status and tumor size in conjunction with other factors while NATALEE incorporates node-negative patients with specific high-risk features like high genomic risk. Consequently, an inherited higher risk is prominent for MonarchE compared to NATALEE criteria.

**Table 1:** Defined high-risk patients between clinical trials with CDK4/6 inhibitor for early hormone receptor (HR)+/human epidermal growth factor receptor II (HER2)-breast cancers

Criteria	Natalee trial	MonarchE trial
Nodal status	Node-negative (N0) with high-risk features, or node-positive	4 or more positive lymph nodes, or 1–3 positive lymph nodes with additional high-risk features
Tumor size	Not specified	Tumor size ≥ 5 cm for patients with 1–3 positive lymph nodes, or tumor size < 5 cm with additional high-risk features
Grade	N0 with Grade 2 and high-risk features (e.g., Ki67 ≥ 20%, or high genomic risk profile, or Oncotype Dx ≥ 26) or N0 with Grade 3	Grade 3 for patients with 1–3 positive lymph nodes, or Grade < 3 with additional high-risk features
Ki67 Index	Ki67 ≥ 20% for node-negative patients with Grade 2	Ki67 ≥ 20% for patients with 1–3 positive lymph nodes, or Ki67 < 20% with additional high-risk features
Genomic risk profile	High genomic risk profile (e.g., Oncotype DX ≥ 26) for node-negative patients with Grade 2	Not specified

This study is novel in its use of real-world data from a Taiwanese institution to compare the prognostic value of MonarchE and NATALEE trial criteria in HR+/HER2− early breast cancer patients, evaluating the impact of adjuvant chemotherapy and providing a detailed stratification of survival outcomes across combined risk groups; importantly it also performs a post-hoc analysis that reveals stage III N-high patients benefit from chemotherapy.

## Limitations

This study utilized cancer registry data to provide real-world evidence, but its retrospective design was associated with potential limitations, such as selection bias, loss to follow-up, and data inaccuracies. While Taiwan’s cancer registry is recognized for its high reliability, with an accuracy rate exceeding 95% [33], the follow-up period (8 years) may be insufficient to fully assess late recurrences, a common concern in HR+/HER2− EBC [16, 34, 35]. The results were drawn from a single institution in Taiwan, which may limit their applicability to broader, ethnically diverse populations. Additionally, the study period predated the introduction of CDK4/6i, preventing an evaluation of its interaction with chemotherapy.

## Conclusion

Using a single-institution’s cancer registry for Taiwanese HR+/HER2− early breast cancers, we demonstrated that both the NATALEE and MonarchE high-risk criteria were prognostic. Adjuvant chemotherapy significantly reduced recurrence and mortality risks among M-H patients. For the N-H high-risk population, only those with pathological stage III benefited from chemotherapy. Further studies are needed to explore the interplay between chemotherapy and CDK4/6i in high-risk populations.

## Supplementary Information

Below is the link to the electronic supplementary material.

Supplementary file1 (DOCX 107 KB)

## Data Availability

All data generated or analysed during this study are included in this published article [and its supplementary information files].
