# Supplementary material for: Survival outcomes in trial defined high-risk hormone receptor-positive/human epidermal growth factor receptor II-negative early breast cancer: impact of adjuvant chemotherapy
Source: Discov Oncol. 2025 May 16;16:783. doi: 10.1007/s12672-025-02601-4 (PMC12084447; doi:10.1007/s12672-025-02601-4)
Supplement: Supplementary file 1 — Supplementary file1 (DOCX 107 KB) [file 12672_2025_2601_MOESM1_ESM.docx]

**Supplementary materials**

**Supplementary Figures**


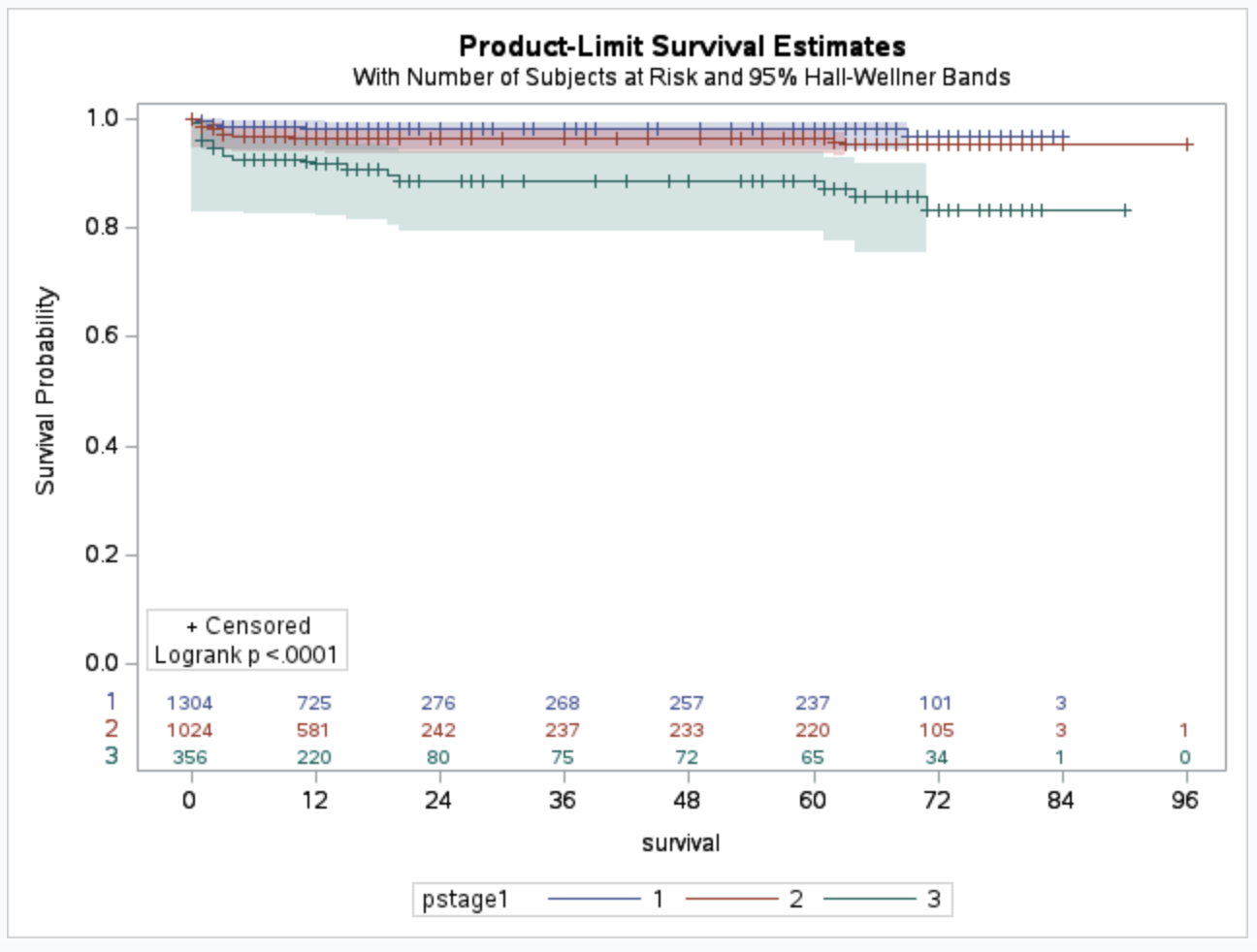


**Supplementary Figure 1.** Recurrence-free survival across pathological stages I, II, and III (log-rank test: P-value<0.001)

**Supplementary Table 1. Summary of survival outcomes.**

| Risk Group | 8-year OS Rate (%) | Mean OS (Years) | 8-year RFS Rate (%) | Mean RFS (Years) | Key findings |
| --- | --- | --- | --- | --- | --- |
| N-H (All) | 96 | 5.6 | - | - | HR = 2.3 vs. N-L |
| N-L (All) | 98.7 | 6.7 | - | - |  |
| M-H (All) | - | 5.3 | - | - | HR = 2.8 vs. M-L |
| M-L (All) | - | 6.7 | - | - |  |
| N-H/M-H | 94.8 | 6.7 | 95.5 | 5.4 |  |
| N-H/M-L | 96.8 | 6.7 | 96.9 | 5.7 |  |
| N-L/M-H | 90.6 | 6.7 | 94 | 3.2 | Poorest OS |
| N-L/M-L | 98.9 | 6.7 | 98.9 | 6.7 | Best outcomes |
| N-H with chemo | 96.6 | 6.7 | 94.5 | - | OS: NS benefit |
| N-H without chemo | 93.4 | 6.7 | 96 | - | RFS: no benefit |
| M-H with chemo | 95.5 | 6.7 | 93.3 | - | OS & RFS Benefit |
| M-H without chemo | 71.4 | 6.7 | 81.3 | - |  |
| N-H Stage III w/chemo | - | 6.7 | 92.4 | - | RFS Benefit |
| N-H Stage III w/o chemo | - | 6.7 | 72.7 | - |  |

(OS: overall survival, RFS = recurrence-free survival, N-H: NATALEE high-risk, N-L: NATALEE low-risk, M-H: MonarchE high-risk, M-L: MonarchE low-risk, chemo: chemotherapy, NS: not significant, HR: hazard ratio)
